# Interaction Effect of EDTA, Salinity, and Oxide Nanoparticles on Alga *Chlamydomonas reinhardtii* and *Chlamydomonas euryale*

**DOI:** 10.3390/plants10102118

**Published:** 2021-10-06

**Authors:** Emilie Canuel, Cleiton Vaz, William Gerson Matias, David Dewez

**Affiliations:** 1 Laboratory of Environmental & Analytical Biochemistry of Contaminants, Department of Chemistry, University of Quebec at Montreal, CP 8888, Succ. Centre-Ville, Montréal, QC H3C 3P8, Canada; emilie.canuel1@gmail.com; 2 Centro Universitário—Católica de Santa Catarina, Rua Visconde de Taunay, 427, Joinville CEP-89203-005, SC, Brazil; contato.cleitonvaz@gmail.com; 3 Laboratório de Toxicologia Ambiental—LABTOX, Departamento de Engenharia Sanitária e Ambiental, Campus Universitário, Universidade Federal de Santa Catarina, Florianópolis CEP-88040-970, SC, Brazil; william.g.matias@ufsc.br

**Keywords:** EDTA, salinity, Fe_3_O_4_, CuO, nanoparticles, *C. reinhardtii*, *C. euryale*

## Abstract

The interaction effects of organic ligand ethylene diamine tetra-acetic acid (EDTA) and oxide nanoparticles (magnetite Fe_3_O_4_-NPs and copper CuO-NPs) were investigated during a 72 h period on two green algal species—*Chlamydomonas reinhardtii* under freshwater conditions and *Chlamydomonas euryale* under saltwater conditions. Fe_3_O_4_-NPs had larger agglomerates and very low solubility. CuO-NPs, having smaller agglomerates and higher solubility, were more toxic than Fe_3_O_4_-NPs in freshwater conditions for similar mass-based concentrations, especially at 72 h under 100 mg L^−1^. Furthermore, the effect of EDTA increased nanoparticle solubility, and the salinity caused a decrease in their solubility. Our results on *C. euryale* showed that the increase in salinity to 32 g L^−1^ caused the formation of larger nanoparticle agglomerates, leading to a decrease in the toxicity impact on algal cells. In addition, EDTA treatments induced a toxicity effect on both freshwater and saltwater *Chlamydomonas* species, by altering the nutrient uptake of algal cells. However, *C. euryale* was more resistant to EDTA toxicity than *C. reinhardtii*. Moreover, nanoparticle treatments caused a reduction in EDTA toxicity, especially for CuO-NPs. Therefore, the toxicity impact caused by these environmental factors should be considered in risk assessment for metallic nanoparticles.

## 1. Introduction

Nanomaterials are widely used in industries for many technological applications. Nanoparticles of magnetite (Fe_3_O_4_-NPs) are applied in magnetic resonance imaging and medical treatments, such as cancer [1] and in wastewater treatment [2,3,4]. Nanoparticles of copper oxide (CuO-NPs) are in fabrics and electronic products, providing antimicrobial and thermal conductivity properties [5,6]. In the long-term, massive production of these nanomaterials may represent a risk of contamination for aquatic environments, from their manufacturing into consumer products to their utilization and degradation [7].

It was previously suggested that the toxicity mechanisms of metallic NPs were dependent on their physicochemical properties [8,9,10,11]. Several studies investigated the toxicity effects of Fe_3_O_4_-NPs and CuO-NPs on a wide variety of microorganisms, such as bacteria *Escherichia coli*, *Bacillus subtilis*, *Vibrio fischeri* and *Streptococcus aureus* [12,13], cyanobacteria *Microcystis aeruginosa* [14], microalgae *Chlorella vulgaris*, *Pseudokirchneriella subcapitata*, *Chlamydomonas reinhardtii*, *Chlorella pyrenoidosa* and *Coelastrella terrestris* [13,15,16,17,18,19,20], and picoplankton *Picochlorum* sp. [21]. These studies focused on the cellular toxicity impact in a concentration–response relationship, related to the time of exposure. However, they did not consider the interaction effect of environmental parameters on the physicochemical and toxicity properties of tested NPs. Indeed, these properties might change due to abiotic conditions, such as light intensity, temperature degree, pH, salinity level, or organic ligands. Only one study previously investigated the effect of light on the toxicity of CuO-NPs by monitoring biological endpoints of the alga *C. reinhardtii* [22]. The authors demonstrated that the exposure to 0.8 mg L^−1^ of CuO-NPs combined with light-enhanced UVB radiation caused a synergistic toxicity impact on algal cells.

Ethylene diamine tetra-acetic acid (EDTA), known as a strong organic ligand, is widely used in detergents, wastewater treatment, pulp and paper industry, food products, and metal phytoremediation [23,24,25]. High concentrations of EDTA were found in rivers, showing the persistence of this compound in aquatic environments [24,26,27]. Ma et al. (2003) [28] showed that EDTA and fulvic acid significantly decreased the toxicity of copper on *Scenedesmus subspicatus* by complexing Cu ions. Another study on the macroalga *Gracilaria domingensis* showed that the toxicity effects of EDTA complexes with cadmium, copper, zinc, and lead were significantly lower compared to free metals [29]. Recently, Pascual et al. (2020) [30] demonstrated that the presence of EDTA in media was important to control the toxicity of copper, zinc, and lead on the growth of alga *P. subcapitata*. Moreover, it was suggested that organic ligands would tend to stabilize metallic NPs agglomerations (ZnS and Ag), from both electrostatic and steric effects [31,32,33]. However, the effect of organic ligands on the toxicological properties of non-functionalized metallic NPs was little investigated. One study previously reported that the fulvic acid of the Suwannee River increased the toxicity of CuO-NPs on the freshwater cyanobacteria *Microcystis aeruginosa*, by increasing the dissolution of Cu ions and their absorption through the cell wall [14]. Therefore, more studies need to be conducted concerning the effect of organic ligands, such as EDTA, on the properties of metallic NPs and their combined toxicity effects on organisms.

In this study, the main objective was to investigate the interaction effect of environmental factors on the growth and viability of two green alga species—*Chlamydomonas reinhardtii* under freshwater conditions, and *Chlamydomonas euryale* under saltwater conditions. In fact, strains of the *Chlamydomonas* genus were widely used as model organisms in laboratory studies of stress conditions on algal physiology [34,35]. Here, the marine species *C. euryale* was, for the first time, used in abiotic stress toxicity testing. As environmental factors, we tested the change of salinity level (10 and 32 g L^−1^), the presence of organic ligand EDTA (10 and 100 mg L^−1^), and the effect of Fe_3_O_4_-NPs and CuO-NPs (50 and 100 mg L^−1^). Therefore, the toxicity impact on algal cells is discussed according to the change of these environmental factors and their interactions.

## 2. Results and Discussion

### 2.1. Effect of Salinity and EDTA on NPs Properties

**Figure 1:**
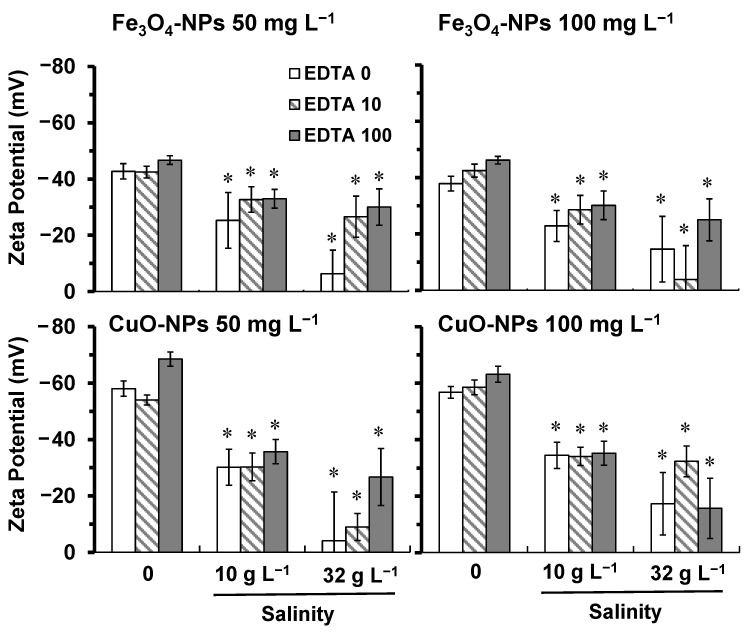
Zeta potential values of NPs in HSM (no salt added), and under two salinity conditions (10 and 32 g L^−1^). NPs were also exposed to EDTA concentrations: 0 mg L^−1^ (white), 10 mg L^−1^ (dash) and 100 mg L^−1^ (gray). (*) For both NPs, the salinity significantly decreased (*p* < 0.05) the values of the zeta potential compared to the respective controls (no salt added), even in the presence of EDTA (determined by one-way ANOVA and Tukey post-hoc test).

In this study, we characterized NPs in suspension to determine the surface charge, the average hydrodynamic diameter, and the solubility property in relation to the change of salinity level and the concentration of EDTA. The obtained results showed that the chemical properties of both NPs in modified high salt medium (HSM) changed similarly related to the salinity level. Indeed, a stronger salinity level induced a higher ionic strength in the medium. The HSM freshwater condition had an ionic strength of 1.75 × 10^−2^ eq L^−1^, and the HSM with salinities of 10 and 32 g L^−1^ had 1.043 × 10^−1^ and 2.914 × 10^−1^ eq L^−1^, respectively. The results on zeta potential indicated that the ionic strength of HSM induced a negative surface charge to both NPs, and the charge intensity was higher for CuO-NPs compared to Fe_3_O_4_-NPs (Figure 1).

**Figure 2:**
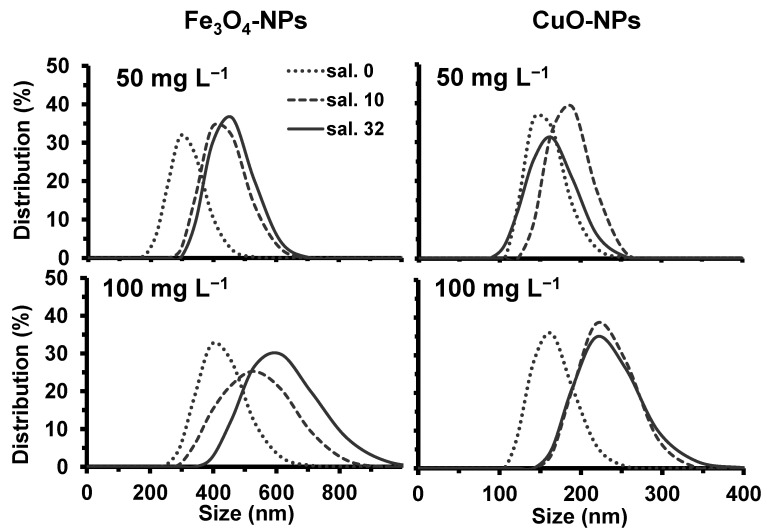
Hydrodynamic diameter distribution of Fe_3_O_4_ and CuO particle size in HSM (dots), and under two salinity conditions, 10 g L^−1^ (dash) and 32 g L^−1^ (line).

When comparing all conditions, the surface charge of both NPs showed a decrease in the intensity, which was correlated with the increase of the ionic strength. The results in Figure 1 showed that values of the Zeta potential of both NPs got closer to zero under salinities of 10 and 32 g L^−1^ in comparison to freshwater conditions. Consequently, this neutralizing effect of NPs surface charge caused an increase in NPs agglomeration, as indicated by the change of the hydrodynamic diameter distribution of particle size (Figure 2). The EDTA did not have a significant effect on the distribution of particle size diameters compared to the control for both NPs. Under salinities of 10 and 32 g L^−1^, the average hydrodynamic diameter of particles was 395–460 nm for tested 50 mg L^−1^ of Fe_3_O_4_-NPs (Figure 2), whereas it was 165–190 nm for 50 mg L^−1^ of CuO-NPs (Figure 2). Therefore, these results indicated that a salinity from 10 g L^−1^ significantly induced the formation of larger agglomerates of NPs caused by a higher ionic strength in the medium.

When comparing both NPs in freshwater HSM (Figure 2), CuO-NPs did form smaller agglomerates (100–250 nm) than Fe_3_O_4_-NPs (200–450 nm). In fact, a higher intensity of the surface charge allowed the NPs dispersion to be more stable due to electrostatic repulsion forces. Our results showed that Fe_3_O_4_-NPs had a surface charge of −43.0 ± 2.1 mV, and −59.9 ± 2.4 mV for CuO-NPs (Figure 1). Since the HSM had a neutral pH, the isoelectric point of Fe_3_O_4_-NPs (6.5 ± 0.2) was much lower and near the neutral pH than the isoelectric point of CuO-NPs (9.5 ± 0.4), which explained the lower intensity of the surface charge for Fe_3_O_4_-NPs. Consequently, these zeta potential values explained the difference in the formation of agglomerates between the two NPs.

When comparing the effect of 50 and 100 mg L^−1^ of NPs, the intensity of the surface charge was not significantly affected (Figure 1). However, our results showed that a higher NPs concentration induced the formation of larger agglomerates (Figure 2). From 50 to 100 mg L^−1^ of NPs, the average of Fe_3_O_4_-NPs diameters increased by 100 nm in freshwater HSM (Figure 2), while it increased by 20 nm for CuO-NPs (Figure 2). Indeed, the formation of larger NPs agglomerates is explained here by more particle-particle interactions in the highest concentrated solution (100 mg L^−1^), destabilizing NPs dispersion in the medium [9]. For NPs at 100 mg L^−1^, salinities of 10 and 32 g L^−1^ caused significantly (*p* < 0.05) the formation of larger NPs agglomerates compared to freshwater condition. Our results indicated that, from 50 to 100 mg L^−1^ of NPs, the average of Fe_3_O_4_-NPs diameters increased by 135–160 nm (Figure 2), while it increased by 30–55 nm for CuO-NPs (Figure 2).

**Figure 3:**
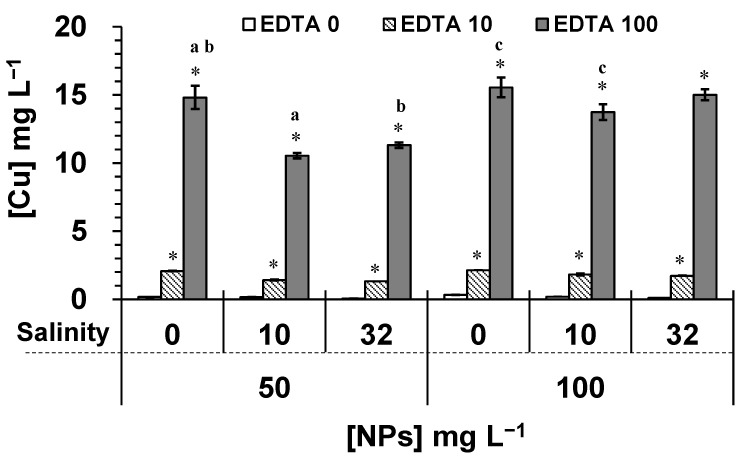
Solubility of CuO-NPs (50 and 100 mg L^−1^) in HSM (no salt added), and under two salinity conditions, 10 g L^−1^ and 32 g L^−1^. NPs were exposed to EDTA concentrations: 0 (white), 10 mg L^−1^ (dash), and 100 mg L^−1^ (gray). (*) The EDTA effect on NPs significantly caused an increase (*p* < 0.05) in the concentration of free Cu compared to respective controls (no EDTA). Moreover, the same letters (a, b, or c) indicated a significant difference for *p* < 0.05 (determined by one-way ANOVA and Tukey post-hoc test).

The solubility of NPs was also determined under various salinity and EDTA concentrations. Fe_3_O_4_-NPs had a very low solubility in freshwater HSM, and the methodology used did not permit to quantify the concentration as being less than the detection limit of the device (<0.02 mg L^−1^). The salinity and EDTA did not significantly affect the solubility of Fe_3_O_4_-NPs, when compared to the control. Considering known properties of metallic NPs in solution [9], the low solubility of Fe_3_O_4_-NPs was explained by the strong aggregation state of primary particles sintered (atoms held by strong chemical bonds), and to the formation of large agglomerates (suspensions held by weak van der Waals forces) reducing surface contact in solution. On the contrary, our results indicated that the solubility of CuO-NPs was correlated with the NPs concentration. The soluble fraction of free Cu released from 50 and 100 mg L^−1^ of CuO-NPs in freshwater HSM (no added salt and no EDTA) was of 0.17 and 0.33 mg L^−1^ per day, respectively (Figure 3). However, the salinity at 32 g L^−1^ caused a lower solubility of CuO-NPs (compared to controls for *p* < 0.05) reaching 0.07 and 0.11 mg L^−1^ per day, respectively, for 50 and 100 mg L^−1^ of CuO-NPs. Under this condition, the salinity caused the formation of larger agglomerates, reducing the surface contact in solution. 

The interaction of EDTA with NPs increased significantly (*p* < 0.05) the solubility of CuO-NPs, which was concentration dependent (Figure 3). When CuO-NPs suspensions were exposed to 100 mg L^−1^ of EDTA in HSM, the soluble fraction of released Cu increased by 87 and 47 times compared to controls (*p* < 0.05) for 50 and 100 mg L^−1^ of CuO-NPs, respectively. The formation of EDTA complexes with metals can be estimated by the constant stability of the complex metal-ligand and the metal concentration [36]. The ionic forms of Cu and Fe are known to have a good affinity with EDTA compounds, since the dissociation constants of EDTA-metal complexes (pKc) are 18.8 for Cu^2+^, 14.3 for Fe^2+^, and 25.1 for Fe^3+^ [37]. However, the effect of EDTA on the solubility property of metallic NPs such as Fe_3_O_4_-NPs and CuO-NPs has not been documented. Only one previous study investigated the effect of humic and fulvic acids on the release of Cu from Cu-NPs in solution [38]. The authors demonstrated that a high concentration of humic substances (100 mg L^−1^) was effective in promoting the dissolution of Cu-NPs during 24 h, and the release of Cu from NPs was able to reach 13.79 mg L^−1^. This phenomenon was explained by the complexation reactions between Cu-NP and the functional groups (carboxylic and/or phenolic) of humic acids. In fact, our results showed a similar effect of EDTA on the solubility of CuO-NPs. The EDTA compounds were able to interact with carboxylic groups of CuO-NPs, and the release of copper would remain solubilized as a complex EDTA-Cu or as free Cu^2+^ in solution. Therefore, our results suggested that the lack of chemical interaction between EDTA and Fe_3_O_4_-NPs was caused by its more complex atomic structure (aggregation state) compared to the simple oxide CuO-NPs. At high salinity (32 g L^−1^), the effect of 100 mg L^−1^ EDTA caused a release of 11.31 and 15.01 mg L^−1^ of Cu, respectively, for 50 and 100 mg L^−1^ of CuO-NPs. However, under this EDTA treatment, the concentration of released Cu significantly decreased by 29% and 24% when compared to HSM (no added salt for *p* < 0.05) for 50 mg L^−1^ of CuO-NPs under salinities of 10 g L^−1^ and 32 g L^−1^, respectively. For 100 mg L^−1^ of CuO-NPs, the concentration of released Cu significantly decreased by 12% only under salinity of 10 g L^−1^ (Figure 3).

### 2.2. Toxicity Testing on Algal Cells

**Figure 4:**
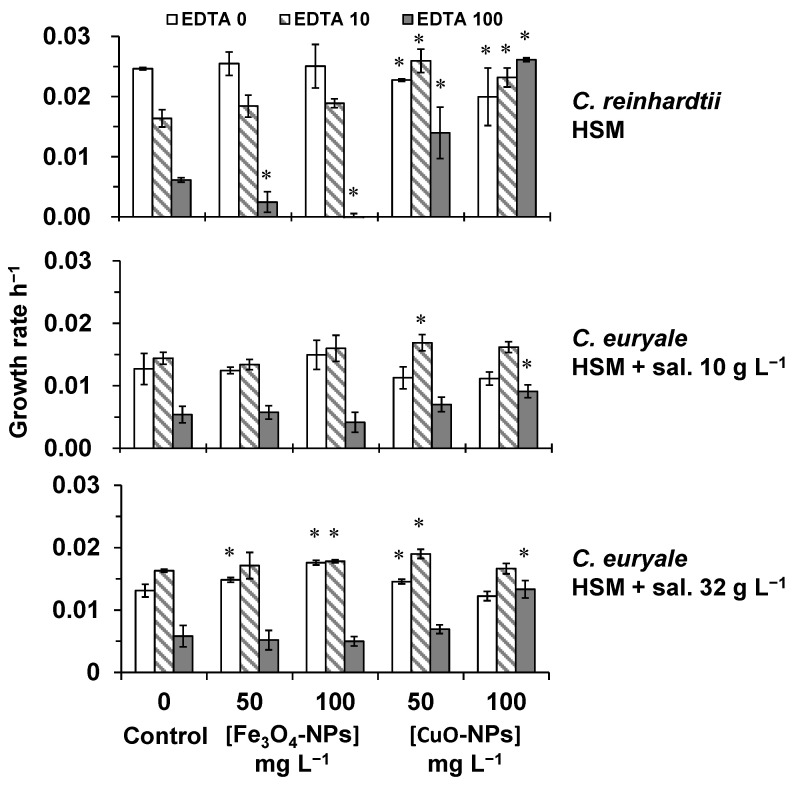
Growth rate (h^−1^) of algal cells exposed during 72 h to 50 and 100 mg L^−1^ of Fe_3_O_4_-NPs or CuO-NPs for *C. reinhardtii* in HSM, and *C. euryale* in HSM under two salinity conditions, 10 g L^−1^ and 32 g L^−1^. Tested concentrations of EDTA: 0 mg L^−1^ (white), 10 mg L^−1^ (dash) and 100 mg L^−1^ (gray). (*) Significant differences between the controls and treatments (*p* < 0.05) were determined by one-way ANOVA and Tukey post-hoc test.

The effects of Fe_3_O_4_-NPs and CuO-NPs was investigated during 72 h on algal cells of *C. reinhardtii* and *C. euryale*, and under different EDTA concentrations (Figure 4). The growth rate of *C. reinhardtii* exposed to Fe_3_O_4_-NPs (without EDTA) was not significantly affected in comparison to the control, but algal cells exposed to CuO-NPs at 50 and 100 mg L^−1^ showed a significant decrease (*p* < 0.05) by 8 and 19%, respectively (Figure 4). In addition, the EDTA induced a significant effect on the growth rate of *C. reinhardtii* in HSM compared to control (no EDTA and no NPs treatments). This inhibitory effect was of 34 and 75% (compared to control) under EDTA treatments of 10 and 100 mg L^−1^, respectively (Figure 4). However, the combined effect of EDTA and NPs treatments was different for Fe_3_O_4_-NPs and CuO-NPs. Under high EDTA treatment (100 mg L^−1^), the growth rate of *C. reinhardtii* exposed to Fe_3_O_4_-NPs at 50 and 100 mg L^−1^ significantly decreased (*p* < 0.05) by 60 and 100%, respectively. On the other hand, the toxic effect of EDTA on the growth rate of *C. reinhardtii* was attenuated or completely neutralized by CuO-NPs in HSM (Figure 4). This phenomenon was explained by the high EDTA affinity to react with CuO-NPs suspensions in HSM, leaving algal cells free from EDTA effect.

The growth rate of *C. euryale* exposed to Fe_3_O_4_-NPs or CuO-NPs was not significantly inhibited (compared to controls, *p* < 0.05) in HSM with salinities of 10 and 32 g L^−1^, even under EDTA effect (Figure 4). Only the effect of high EDTA concentration alone (100 mg L^−1^) induced a significant decrease (*p* < 0.05) in the growth rate of *C. euryale* by 56–58% compared to controls. It was interesting to notice that this inhibitory effect was weaker compared to the effect on *C. reinhardtii* in HSM (75%), indicating that *C. euryale* was less sensitive to the toxicity of EDTA probably because of the salinity level in the media in which EDTA molecules formed complexes with sodium.

**Figure 5:**
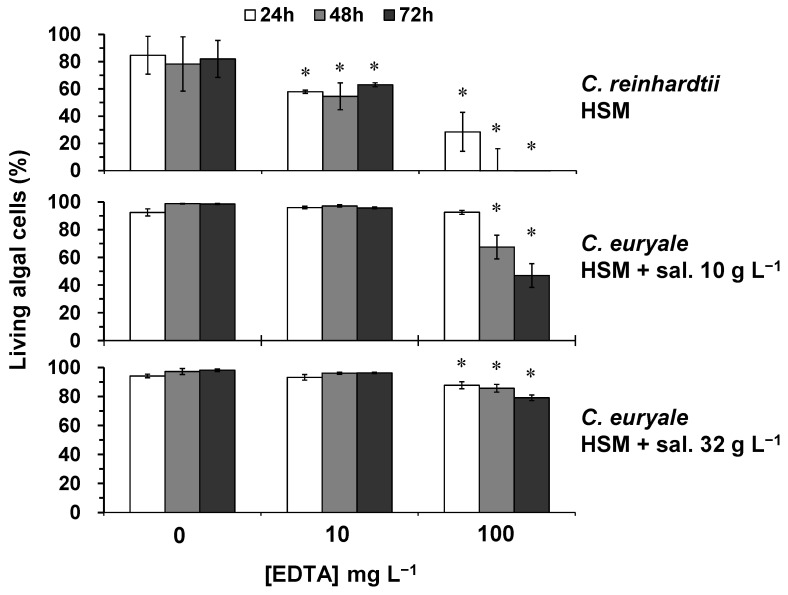
Living algal cells (%) at 24, 48, and 72 h of exposure to different concentrations of EDTA (0, 10 and 100 mg L^−1^) for *C. reinhardtii* in HSM, and *C. euryale* in HSM under two salinity conditions, 10 g L^−1^ and 32 g L^−1^. (*) Significant differences between the controls and treatments (*p* < 0.05) were determined by one-way ANOVA and Tukey post-hoc test.

The obtained results showed that EDTA treatments during 24–72 h caused a significant decrease in the proportion of *C. reinhardtii* living cells (*p* < 0.05), which was dependent to the EDTA concentration and the time of exposure (Figure 5). The cellular mortality was by 23–32% under 10 mg L^−1^ of EDTA during 24–72 h. When algal cells were exposed to 100 mg L^−1^ of EDTA, the cellular mortality was by 66% at 24 h and reaching 100% at 48–72 h. This toxicity effect indicated that EDTA molecules were trapping essential elements causing a deficiency in nutrient uptake at the surface of algal cells, as it was previously suggested [30,39]. On the other hand, only 100 mg L^−1^ of EDTA induced a significant toxicity effect (*p* < 0.05) on the cellular viability of *C. euryale* (Figure 5). Under a salinity of 10 g L^−1^, the cellular mortality was of 30 and 52% at 48 and 72 h, respectively. Under a salinity of 32 g L^−1^, it was only of 7, 12, and 20% at 24, 48, and 72 h, respectively. These results are in accordance with the change of growth rate, indicating that the increase of salinity level significantly reduced the toxicity effect of EDTA on algal cells.

**Figure 6:**
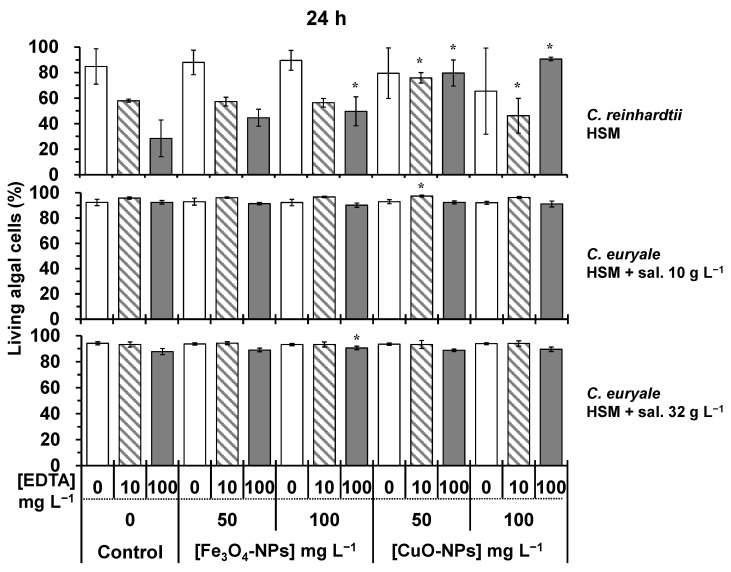
Living algal cells (%) at 24 h for the control and treated cells to 50 and 100 mg L^−1^ Fe_3_O_4_-NPs or CuO-NPs: *C. reinhardtii* in HSM; *C. euryale* in HSM under two salinity conditions, 10 g L^−1^ and 32 g L^−1^. Under these conditions, algal cells were exposed to EDTA concentrations: 0 (white), 10 mg L^−1^ (dash), and 100 mg L^−1^ (gray). (*) Significant differences between the controls and treatments (*p* < 0.05) were determined by one-way ANOVA and Tukey post-hoc test.

**Figure 7:**
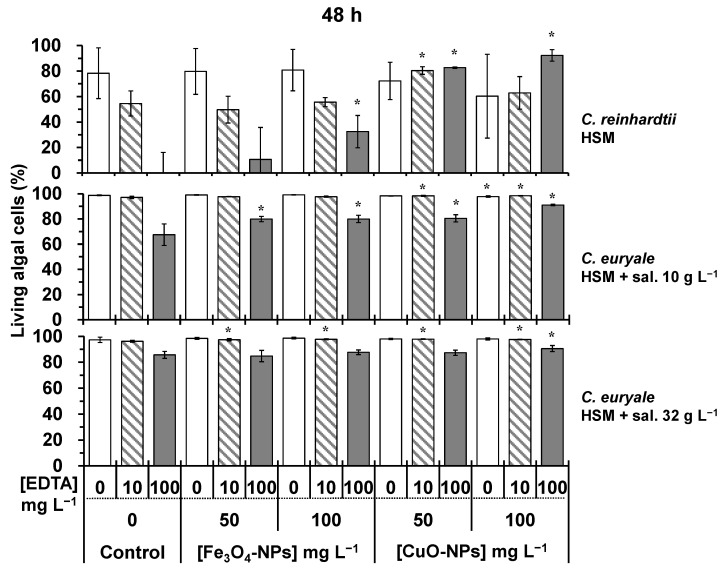
Living algal cells (%) at 48 h for the control and treated cells to 50 and 100 mg L^−1^ Fe_3_O_4_-NPs or CuO-NPs: *C. reinhardtii* in HSM; *C. euryale* in HSM under two salinity conditions, 10 g L^−1^ and 32 g L^−1^. Under these conditions, algal cells were exposed to EDTA concentrations: 0 (white), 10 mg L^−1^ (dash), and 100 mg L^−1^ (gray). (*) Significant differences between the controls and treatments (*p* < 0.05) were determined by one-way ANOVA and Tukey post-hoc test.

**Figure 8:**
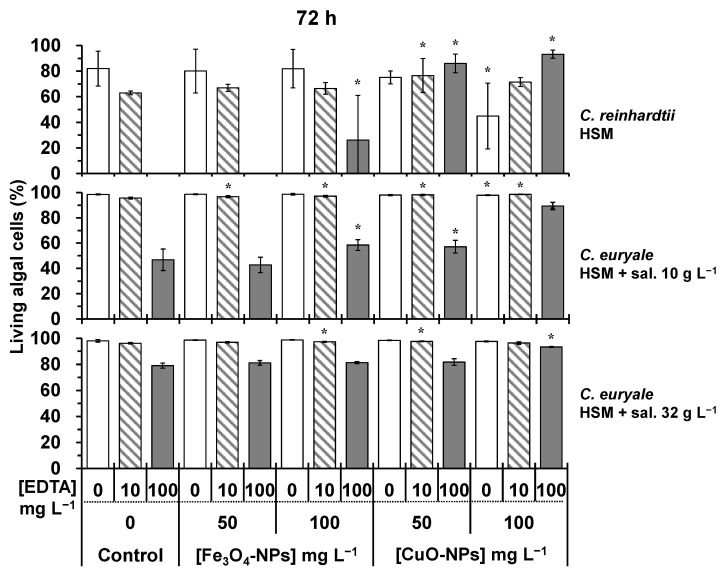
Living algal cells (in %) at 72 h for the control and treated cells to 50 and 100 mg L^−1^ Fe_3_O_4_-NPs or CuO-NPs: *C. reinhardtii* in HSM; *C. euryale* in HSM under two salinity conditions, 10 g L^−1^ and 32 g L^−1^. Under these conditions, algal cells were exposed to EDTA concentrations: 0 (white), 10 mg L^−1^ (dash), and 100 mg L^−1^ (gray). (*) Significant differences between the controls and treatments (*p* < 0.05) were determined by one-way ANOVA and Tukey post-hoc test.

Without EDTA treatments, the cellular viability of *C. reinhardtii* and *C. euryale* exposed during 24–72 h to Fe_3_O_4_-NPs did not significantly decrease (*p* < 0.05) in comparison to controls (Figure 6, Figure 7 and Figure 8). It appeared that these Chlamydomonas species were more resistant to the toxic effects of Fe_3_O_4_-NPs than the previous studied green alga *Chlorella vulgaris*, and this was probably due to its cellular characteristics such as the cell size and morphology [16]. In this work, the authors showed that the cellular viability decreased by 45 and 60%, when algal cells of *C. vulgaris* were exposed during 72 h to 50 and 100 mg L^−1^ of Fe_3_O_4_-NPs, respectively. Furthermore, the combined effect of EDTA and Fe_3_O_4_-NPs treatments did not cause a higher cellular mortality on *C. reinhardtii* and *C. euryale* during 24–72 h. Even under some conditions, the effect of EDTA was less strong under Fe_3_O_4_-NPs treatments, showing an antagonistic effect of this NP on EDTA toxicity (Figure 6, Figure 7 and Figure 8). In particular, the inhibitory effect of EDTA on the cellular viability of *C. reinhardtii* was respectively lower compared to the control (no NPs) by 43%, 32%, 26% at 24, 48, and 72 h (*p* < 0.05), under 100 mg L^−1^ of Fe_3_O_4_-NPs. Under a salinity of 10 g L^−1^, the inhibitory effect of EDTA on the cellular viability of *C. euryale* exposed to 100 mg L^−1^ of Fe_3_O_4_-NPs was lower by 16% and 20% at 48 and 72 h, respectively (*p* < 0.05).

Without EDTA treatments, the cellular viability of *C. reinhardtii* and *C. euryale* was significantly affected (*p* < 0.05) only under 100 mg L^−1^ of CuO-NPs. For *C. reinhardtii*, this parameter decreased by 45 % at 72 h, and it slightly decreased (1–2%) for *C. euryale* at 48 and 72 h only under a salinity of 10 g L^−1^ (Figure 7 and Figure 8). Furthermore, the combination of EDTA and CuO-NPs treatments did not induce an additive effect on the cellular mortality of *C. reinhardtii* and *C. euryale* during 24–72 h. During 24–72 h, the toxicity effect of EDTA was counterbalanced by 50 and 100 mg L^−1^ of CuO-NPs (Figure 6, Figure 7 and Figure 8).

## 3. Conclusions

The interaction effect of environmental factors (EDTA, oxide NPs and/or salinity-depending on the species) was investigated during 72 h on the growth and viability of *Chlamydomonas reinhardtii* under freshwater condition, and *Chlamydomonas euryale* under saltwater conditions. Due to lower particles agglomeration and higher solubility, CuO-NPs were more toxic than Fe_3_O_4_-NPs in freshwater condition, especially at 72 h under high concentration (100 mg L^−1^). Even though the specific surface area of CuO-NPs was twice smaller than Fe_3_O_4_-NPs, it was important to notice that the toxicity of CuO-NPs was stronger than Fe_3_O_4_-NPs for similar mass-based concentration. Indeed, Fe_3_O_4_-NPs had larger agglomerates and a very low solubility. Furthermore, the effect of EDTA increased the solubility of NPs, and the salinity caused a decrease in their solubility. Our results on *C. euryale* showed that the increase in the salinity level caused the formation of larger NPs agglomerates, leading to a decrease in the toxicity impact on algal cells. The increase of agglomerates could induce a lower bioavailability of NPs to interact with algal cells, and most of these agglomerates would sediment. Therefore, Fe_3_O_4_-NPs and CuO-NPs would be less dangerous in marine environments compared to freshwater ones. Moreover, our results showed that EDTA treatments induced a cellular toxicity effect on both freshwater and saltwater *Chlamydomonas* species, indicating the property of EDTA to alter the nutrient uptake at the surface of algal cells. Toxicity testing during 72 h demonstrated that algal cells of *C. euryale* were more resistant to EDTA toxic effects than algal cells of *C. reinhardtii*. In addition, our results indicated that NPs treatments induced an antagonistic effect on EDTA toxicity, especially for CuO-NPs. The marine condition (salinity level of 32 g L^−1^) also caused a decrease in the toxicity of EDTA on algal cells. In this perspective, more studies need to be done on chronic toxicity testing of these stressors to determine the long-term impact on algal population. Although the contamination of EDTA and oxide NPs is not alarming at a very low concentration, recent data in situ are missing for both freshwater and marine environments. Nevertheless, the toxicity impact caused by the interaction effect of environmental factors (EDTA and salinity) should be considered in risk assessment for metallic NPs.

## 4. Materials and Methods

### 4.1. Algal Strains and Culture

The freshwater alga *Chlamydomonas reinhardtii* (CC-125) was provided by the Chlamydomonas Resource Center (The University of Minnesota, St. Paul, MN 55108, USA), and the marine species *Chlamydomonas euryale* (UTEX 2274) was provided by the UTEX Culture Collection of Algae (The University of Texas at Austin, Austin, TX 78712, USA). Both strains were grown in modified high salt medium (HSM), aka Sueoka’s medium [40]: NH_4_Cl 500 μg mL^−1^; MgSO_4_∙7H_2_O 20 μg mL^−1^; CaCl_2_ 10 μg mL^−1^; KH_2_PO_4_ 740 μg mL^−1^; K_2_HPO_4_ 1.44 mg mL^−1^; H_3_BO_3_ 185.5 μg mL^−1^; MnCl_2_∙4H_2_O 415.4 μg mL^−1^; ZnCl_2_ 3.3 μg mL^−1^; FeCl_3_∙6H_2_O 159.8 μg mL^−1^; Na_2_EDTA∙2H_2_O 300.0 μg mL^−1^; CoCl_2_∙6H_2_O 2.6 μg mL^−1^; Na_2_MoO_4_∙2H_2_O 7.3 μg mL^−1^; CuCl_2_∙2H_2_O 0.012 μg mL^−1^. The pH was adjusted to 7.0 ± 0.1 using 1N NaOH. All media were sterilized either in autoclave or by filtration (0.22 μm). The marine strain *C. euryale* could not survive in freshwater HSM, thus the salinity level was adjusted by adding NaCl at 10 and 32 g L^−1^. The ionic strength of culture media was estimated using the software MINEQL+ [41]. Algal cultures were maintained continuously on a rotating shaker (80 rpm), under a light intensity of 100 ± 20 μmol of photons m^−2^ s^−1^, and a temperature of 23 ± 1 °C. Samples of algal cultures in their exponential growth phase were used for analysis. 

### 4.2. Characterization of Nanoparticles

Fe_3_O_4_-NPs were purchased from MTI Corporation (Richmond, VA, USA), having a purity of 99.9%, a size of 20 nm, a spherical shape, and a specific surface area >60 m^2^/g. CuO-NPs were obtained from Aldrich (Cat. 544868; Darmstadt, Germany), having a size <50 nm, a spherical shape, and a specific surface area of 29 m^2^/g. Before the characterization, NPs were dispersed in solution using ultrasonication (probe 1/4″, Fisher Scientific, Waltham, MA, USA) at 30% of power during 2 min, and then suspensions were incubated during 1 h at room temperature.

The Zeta potential of NPs was measured with a ZetaPlus (BrookHaven Instrument Corp., Long Island, NY, USA), and the hydrodynamic size distribution using a Zetasizer Nano S90 (DLS, Malvern, Worcestershire, UK). To determine the solubility of NPs, suspensions were incubated in HSM during 24 h, and then centrifuged at a speed of 12,000 g during 30 min (centrifuge J2-HC, JA-20 rotor, Beckman, Fullerton, CA, USA). The supernatant was collected, and the Cu or Fe in the soluble fraction was quantified by optical emission spectrometry (ICP-OES, model 5100, Agilent Technologies, Santa Clara, CA, USA). Prior to this analysis, the supernatant was examined using a Zetasizer Nano S90 to verify that the sample was free of NPs.

### 4.3. Nanoparticles and EDTA Treatments

Culture of algal cells having an initial cell density of 500,000 cells mL^−1^ were exposed to 50 and 100 mg L^−1^ of CuO-NPs or Fe_3_O_4_-NPs during 72 h in a final volume of 20 mL of medium. Alga *C. euryale* was exposed to NPs under salinity conditions of 10 and 32 g L^−1^, and *C. reinhardtii* only in freshwater HSM. Under these conditions, the effect of EDTA was tested at 10 and 100 mg L^−1^. The EDTA stock solution was of 400 mg L^−1^, and the pH was adjusted to the pH of the medium, 7 for HSM and 8.1 for HSM with salinities of 10 and 32 g L^−1^. In this study, we used gravimetric units for CuO and Fe_3_O_4_ NPs characterization, since NPs had properties totally different from their bulk counterpart. Indeed, nanotoxicity testing studies still employed mass-based concentration in water quality standards to discriminate toxicological properties of NPs versus their dissolved ionic species [42].

### 4.4. Growth Rate and Cellular Viability

The change in the cell density was determined by using a Multisizer Z3 (Beckman Coulter Inc., Fullerton, CA, USA), and the growth rate (GR) was calculated according to this formula: GR = (lnW_72h_ − lnW_0_)/72 h, where W_72h_ represents the cell density at 72 h, and W_0_ the initial cell density. Cellular viability was assessed using propidium iodide at 40 μM (as a cellular mortality marker) by flow cytometry (BD Accuri C6, Becton, Dickinson & co., Franklin Lakes, NJ, USA). Results of cellular viability were presented as the proportion of living cells (in %).

### 4.5. Statistical Analysis

All experiments were conducted in two testing series and in triplicate. The one-way analysis of variance (ANOVA) followed by the Tukey post-hoc test was conducted using Origin Pro 9 (2016) Graphing and Analysis software. Significant differences between the controls and treatments were considered for *p* values less than 0.05 (*p* < 0.05).

## Data Availability

All of the results are presented in the article. The raw data and calculation files are available from the corresponding author David Dewez.

## References

[1] Pankhurst Q.A., Thanh N.T.K., Jones S.K. (2009). Progress in applications of magnetic nanoparticles in biomedicine. J. Phys. D.

[2] Panneerselvam P., Morad N., Tan K.A. (2011). Magnetic nanoparticle (Fe_3_O_4_) impregnated onto tea waste for the removal of nickel (II) from aqueous solution. J. Hazard. Mater..

[3] Tang W.W., Zeng G.M., Gong J.L. (2014). Impact of humic/fulvic acid on the removal of heavy metals from aqueous solutions using nanomaterials: A review. Sci. Total Environ..

[4] Huang Y., Keller A.A. (2015). EDTA functionalized magnetic nanoparticle sorbents for cadmium and lead contaminated water treatment. Water Res..

[5] Hwang Y., Park H.S., Jung W.H. (2006). Thermal conductivity and lubrication characteristics of nanofluids. Cur. Appl. Phys..

[6] Dastjerdi R., Montazer M. (2010). A review on the application of inorganic nano-structured materials in the modification of textiles: Focus on anti-microbial properties. Colloids Surf. B.

[7] Gottschalk F., Nowack B. (2011). The release of engineered nanomaterials to the environment. J. Environ. Monitor..

[8] Klaine S.J., Alvarez P.J.J., Batley G.E., Fernandes T.F., Handy R.D., Lyon D.Y., Mahendra S., McLaughlin M.J., Lead J.R. (2008). Nanomaterials in the environment: Behavior, fate, bioavailability, and effects. Environ. Toxicol. Chem..

[9] Jiang J., Oberdörster G., Biswas P. (2009). Characterization of size, surface charge, and agglomeration state of nanoparticle dispersions for toxicological studies. J. Nanopart. Res..

[10] Bhatt I., Tripathi B.N. (2011). Interaction of engineered nanoparticles with various components of the environment and possible strategies for their risk assessment. Chemosphere.

[11] Vale G., Mehennaoui K., Cambier S., Libralato G., Jomini S., Domingos R.F. (2016). Manufactured nanoparticles in the aquatic environment-biochemical responses on freshwater organisms: A critical overview. Aquat. Toxicol..

[12] Baek Y.W., An Y.J. (2011). Microbial toxicity of metal oxide nanoparticles (CuO, NiO, ZnO, and Sb_2_O_3_) to *Escherichia coli*, *Bacillus subtilis*, and *Streptococcus aureus*. Sci. Total Environ..

[13] Aruoja V., Pokhrel S., Sihtmäe M., Mortimer M., Mädler L., Kahru A. (2015). Toxicity of 12 metal-based nanoparticles to algae, bacteria and protozoa. Environ. Sci. Nano..

[14] Wang Z., Li J., Zhao J. (2011). Toxicity and internalization of CuO nanoparticles to prokaryotic alga *Microcystis aeruginosa* as affected by dissolved organic matter. Environ. Sci. Technol..

[15] Aruoja V., Dubourguier H.C., Kahru A. (2009). Toxicity of nanoparticles of CuO, ZnO and TiO_2_ to microalgae *Pseudokirchneriella subcapitata*. Sci. Total Environ..

[16] Barhoumi L., Dewez D. (2013). Toxicity of superparamagnetic iron oxide nanoparticles on green alga *Chlorella vulgaris*. BioMed Res. Int..

[17] Melegari S.P., Perreault F., Matias W.G. (2013). Evaluation of toxicity and oxidative stress induced by copper oxide nanoparticles in the green alga *Chlamydomonas reinhardtii*. Aquat. Toxicol..

[18] Zhao J., Cao X., Liu X., Wang Z., Zhang C., White J., Xing B. (2016). Interactions of CuO nanoparticles with the algae *Chlorella pyrenoidosa*: Adhesion, uptake, and toxicity. Nanotoxicology.

[19] Saxena P., Sangela V., Harish (2020). Toxicity evaluation of iron oxide nanoparticles and accumulation by microalgae *Coelastrella terrestris*. Environ. Sci. Poll. Res..

[20] Saxena P., Saharan V., Baroliya P.K., Gour V.S., Rai M.K., Harish (2021). Mechanism of nanotoxicity in *Chlorella vulgaris* exposed to zinc and iron oxide. Toxicol. Rep..

[21] Hazeem L., Waheed F., Rashdan S., Bououdina M., Loïc B., Slomianny C., Boukherroub R., Elmeselmani W. (2015). Effect of magnetic iron oxide (Fe_3_O_4_) nanoparticles on the growth and photosynthetic pigment content of *Picochlorum* sp.. Environ. Sci. Poll. Res. Int..

[22] Cheloni G., Marti E., Slaveykova V.I. (2015). Interactive effects of copper oxide nanoparticles and light to green alga *Chlamydomonas reinhardtii*. Aquat. Toxicol..

[23] Brul S., Coote P. (1999). Mode of action and microbial resistance mechanisms. Int. J. Food Microbiol..

[24] Nowack B., VanBriesen J.M., Nowack B., Van Briesen J.M. (2005). Chelating agents in the environment. Biogeochemistry of Chelating Agents.

[25] Evangelou M.W.H., Ebel M., Schaeffer A. (2007). Chelate assisted phytoextraction of heavy metals from soil. Effect, mechanism, toxicity, and fate of chelating agents. Chemosphere.

[26] Bucheli-Witschel M., Egli T. (2001). Environmental fate and microbial degradation of aminopolycarboxylic acids. FEMS Microbiol. Rev..

[27] Reemtsma T., Weiss S., Mueller J., Petrovic M., Gonzalez S., Barcelo D., Ventura F., Knepper T.P. (2006). Polar pollutants entry into the water cycle by municipal wastewater: A European perspective. Environ. Sci. Technol..

[28] Ma M., Zhu W., Wang Z. (2003). Accumulation, assimilation and growth inhibition of copper on freshwater alga (*Scenedesmus subspicatus* 86.81 SAG) in the presence of EDTA and fulvic acid. Aquat. Toxicol..

[29] Mendes L., Zambotti-Villela L., Simas C., Colepicolo P. (2015). Toxicological effects of metal-EDTA/NTA complex formation in a synthetic medium on the macroalga *Gracilaria domingensis*. J. Appl. Phycol..

[30] Pascual G., Sano D., Sakamaki T., Nishimura O. (2020). Effects of chemical interaction of nutrients and EDTA on metals toxicity to *Pseudokirckneriella subcapitata*. Ecotox. Environ. Saf..

[31] Hsu-Kim H., Lau B.L.T. (2008). Precipitation and growth of zinc sulfide nanoparticles in the presence of thiol-containing natural organic ligands. Environ. Sci. Technol..

[32] Deonarine A., Lau B.L.T., Aiken G.R., Ryan J.N., Hsu-Kim H. (2011). Effects of humic substances on precipitation and aggregation of zinc sulfide nanoparticles. Environ. Sci. Technol..

[33] Kennedy A.J., Chappell M.A., Bednar A.J., Ryan A.C., Laird J.G., Stanley J.K., Steevens A. (2012). Impact of organic carbon on the stability and toxicity of fresh and stored silver nanoparticles. Environ. Sci. Technol..

[34] Hanikenne M. (2003). *Chlamydomonas reinhardtii* as a eukaryotic photosynthetic model for studies of heavy metal homeostasis and tolerance. New Phytol..

[35] Harris E.H. (2009). The Chlamydomonas Sourcebook: Introduction to Chlamydomonas and Its Laboratory Use.

[36] Nowack B. (2002). Environmental chemistry of aminopolycarboxylate chelating agents. Environ. Sci. Technol..

[37] Tardat-Henry M., Beaudry J.P. (1992). Chimie Des Eaux..

[38] Wang L.F., Habibul N., He D.Q., Li W.W., Zhang X., Jiang H., Yu H.Q. (2015). Copper release from copper nanoparticles in the presence of natural organic matter. Water Res..

[39] Ward T.J., Rausina G.A., Stonebraker P.M., Robinson W.E. (2002). Apparent toxicity resulting from the sequestering of nutrient trace metals during standard *Selenastrum capricornutum* toxicity tests. Aquat. Toxicol..

[40] Sueoka N., Chiang K.S., Kates J.R. (1967). Deoxyribonucleic acid replication in meiosis of *Chlamydomonas reinhardtii*. Isotopic transfer experiments with a strain producing eight zoospores. J. Mol. Biol..

[41] Schecher W.D., McAvoy D.C. (2003). MINEQL+: A Chemical Equilibrium Modeling System, Version 4.5 for Windows, User’s Manual.

[42] Hull M., Kennedy A.J., Detzel C., Vikesland P., Chappell M.A. (2012). Moving beyond mass: The unmet need to consider dose metrics in environmental nanotoxicology studies. Environ. Sci. Technol..

